# Supplementary material for: Assessing the relationship between menstrual products and reproductive and urogenital tract infections (RUTIs): A systematic review evaluating the evidence and recommendations for future research
Source: PLoS One. 2025 Dec 22;20(12):e0332006. doi: 10.1371/journal.pone.0332006 (PMC12721514; doi:10.1371/journal.pone.0332006)
Supplement: S1 Table — Adapted for Entry into Web of Science. (DOCX) [file pone.0332006.s001.docx]

S1 Table. Full Literature Search Strategy for PubMed. Adapted for Entry into Web of Science

| **Outcome** | **Search String** |
| --- | --- |
| Bacterial Vaginosis | ("Menstrual Hygiene Products"[MeSH] OR "menstrual product*"[MeSH] OR "menstrual napkin*"[Title/Abstract] OR "period product*"[Title/Abstract] OR "feminine hygiene product*"[Title/Abstract] OR "feminine hygiene"[Title/Abstract] OR "feminine product*"[Title/Abstract] OR "feminine napkin*"[Title/Abstract] OR "sanitary pad*"[Title/Abstract] OR "menstrual cup"[Title/Abstract] OR "menses cup"[Title/Abstract] OR "menstruation cup"[Title/Abstract] OR "vaginal cup"[Title/Abstract] OR "menstrual cups"[Title/Abstract] OR "menses cups"[Title/Abstract] OR "menstruation cups"[Title/Abstract] OR "vaginal cups"[Title/Abstract] OR "reusable pad*"[Title/Abstract] OR ("menstrual" AND "cloths") OR ("menses" AND "cloths") OR ("menstruation" AND "cloths") OR ("vaginal" AND "cloths") OR ("menstruation"[Title/Abstract] AND "management"[Title/Abstract]) OR (("menstruation"[Title/Abstract] OR "menstrual"[Title/Abstract]) AND "product*"[Title/Abstract] AND "safety"[Title/Abstract]) OR "catamenia") AND ("Bacterial Vaginosis"[MeSH Terms] OR "bacterial vaginosis"[Title/Abstract] OR "BV"[Title/Abstract] OR "vaginal bacteriosis"[Title/Abstract] OR "Gardnerella"[Title/Abstract] OR "vaginal flora imbalance"[Title/Abstract] OR "vaginal dysbiosis"[Title/Abstract]) |
| Urinary Tract Infections | ("Menstrual Hygiene Products"[MeSH] OR "menstrual product*"[MeSH] OR "menstrual napkin*"[Title/Abstract] OR "period product*"[Title/Abstract] OR "feminine hygiene product*"[Title/Abstract] OR "feminine hygiene"[Title/Abstract] OR "feminine product*"[Title/Abstract] OR "feminine napkin*"[Title/Abstract] OR "sanitary pad*"[Title/Abstract] OR "menstrual cup"[Title/Abstract] OR "menses cup"[Title/Abstract] OR "menstruation cup"[Title/Abstract] OR "vaginal cup"[Title/Abstract] OR "menstrual cups"[Title/Abstract] OR "menses cups"[Title/Abstract] OR "menstruation cups"[Title/Abstract] OR "vaginal cups"[Title/Abstract] OR "reusable pad*"[Title/Abstract] OR ("menstrual" AND "cloths") OR ("menses" AND "cloths") OR ("menstruation" AND "cloths") OR ("vaginal" AND "cloths") OR ("menstruation"[Title/Abstract] AND "management"[Title/Abstract]) OR (("menstruation"[Title/Abstract] OR "menstrual"[Title/Abstract] OR "catamenia"[Title/Abstract]) AND "product*"[Title/Abstract] AND "safety"[Title/Abstract])) AND ("Urinary Tract Infections"[MeSH] OR "UTI"[Title/Abstract] OR "urogenital infections"[Title/Abstract] OR "urinary infection*"[Title/Abstract] OR "bladder infection*"[Title/Abstract]) |
| Sexually Transmitted Infections | ("Menstrual Hygiene Products"[MeSH] OR "menstrual product*"[MeSH] OR "menstrual napkin*"[Title/Abstract] OR "period product*"[Title/Abstract] OR "feminine hygiene product*"[Title/Abstract] OR "feminine hygiene"[Title/Abstract] OR "feminine product*"[Title/Abstract] OR "feminine napkin*"[Title/Abstract] OR "sanitary pad*"[Title/Abstract] OR "menstrual cup"[Title/Abstract] OR "menses cup"[Title/Abstract] OR "menstruation cup"[Title/Abstract] OR "vaginal cup"[Title/Abstract] OR "menstrual cups"[Title/Abstract] OR "menses cups"[Title/Abstract] OR "menstruation cups"[Title/Abstract] OR "vaginal cups"[Title/Abstract] OR "reusable pad*"[Title/Abstract] OR ("menstrual" AND "cloths") OR ("menses" AND "cloths") OR ("menstruation" AND "cloths") OR ("vaginal" AND "cloths") OR ("menstruation"[Title/Abstract] AND "management"[Title/Abstract]) OR (("menstruation"[Title/Abstract] OR "menstrual"[Title/Abstract]) AND "product*"[Title/Abstract] AND "safety"[Title/Abstract]) OR "catamenia") AND ("Sexually transmitted disease"[MeSH Terms] OR "Gonorrhea"[Title/Abstract] OR "Syphilis"[Title/Abstract] OR "Chlamydia"[Title/Abstract] OR "Sexually transmitted infections" [Title/Abstract]) |
| HIV | ("Menstrual Hygiene Products"[MeSH] OR "period product*"[Title/Abstract] OR "feminine hygiene product*"[Title/Abstract] OR "feminine hygiene"[Title/Abstract] OR "feminine product*"[Title/Abstract] OR "feminine napkin*"[Title/Abstract] OR "sanitary pad*"[Title/Abstract] OR "menstrual cup"[Title/Abstract] OR "vaginal cup"[Title/Abstract] OR "menstrual cups"[Title/Abstract] OR "menses cups"[Title/Abstract] OR "vaginal cups"[Title/Abstract] OR "reusable pad*"[Title/Abstract] OR ("menstrual"[All Fields] AND "cloths"[All Fields]) OR ("menses"[All Fields] AND "cloths"[All Fields]) OR ("menstruation"[All Fields] AND "cloths"[All Fields]) OR ("vaginal"[All Fields] AND "cloths"[All Fields]) OR ("menstruation"[Title/Abstract] AND "management"[Title/Abstract]) OR (("menstruation"[Title/Abstract] OR "menstrual"[Title/Abstract]) AND "product*"[Title/Abstract] AND "safety"[Title/Abstract]) OR "catamenia"[All Fields]) AND ("HIV"[MeSH] OR "hiv infections"[MeSH] OR "HIV"[All Fields] OR "Human Immunodeficiency Virus"[All Fields] OR "hiv infections"[All Fields]) |
| Human Papilloma Virus | ("Menstrual Hygiene Products"[MeSH] OR "period product*"[Title/Abstract] OR "feminine hygiene product*"[Title/Abstract] OR "feminine hygiene"[Title/Abstract] OR "feminine product*"[Title/Abstract] OR "feminine napkin*"[Title/Abstract] OR "sanitary pad*"[Title/Abstract] OR "menstrual cup"[Title/Abstract] OR "vaginal cup"[Title/Abstract] OR "menstrual cups"[Title/Abstract] OR "menses cups"[Title/Abstract] OR "vaginal cups"[Title/Abstract] OR "reusable pad*"[Title/Abstract] OR ("menstrual"[All Fields] AND "cloths"[All Fields]) OR ("menses"[All Fields] AND "cloths"[All Fields]) OR ("menstruation"[All Fields] AND "cloths"[All Fields]) OR ("vaginal"[All Fields] AND "cloths"[All Fields]) OR ("menstruation"[Title/Abstract] AND "management"[Title/Abstract]) OR (("menstruation"[Title/Abstract] OR "menstrual"[Title/Abstract]) AND "product*"[Title/Abstract] AND "safety"[Title/Abstract]) OR "catamenia"[All Fields]) AND ("Human Papillomavirus Viruses"[MeSH] OR "Human Papillomavirus"[Title/Abstract] OR "Human Papillomavirus Virus"[Title/Abstract] OR "HPV"[Title/Abstract]) |
| All terms | ( "Menstrual Hygiene Products"[Mesh] OR "menstrual hygiene"[tiab] OR "menstrual product*"[tiab] OR "menstrual materials"[tiab] OR "period product*"[tiab] OR "feminine product*"[tiab] OR "feminine hygiene product*"[tiab] OR "feminine napkin*"[tiab] OR "sanitary pad*"[tiab] OR "sanitary napkin*"[tiab] OR "vaginal cup"[tiab:~1] OR "vaginal cups"[tiab:~1] OR ((menstrual[tiab] OR menses[tiab] OR menstruation[tiab] OR catamenia[tiab] OR "feminine hygiene"[tiab]) AND (cup[tiab] OR cups[tiab] OR cloth*[tiab] OR pad[tiab] OR pads[tiab])) )  AND  ( "HIV"[MeSH] OR "HIV Infections"[MeSH] OR "HIV"[tiab] OR "human immunodeficiency virus*" OR "Syphilis"[Mesh] OR syphilis[tiab] OR "Gonorrhea"[Mesh] OR "Neisseria gonorrhoeae"[Mesh] OR gonorrhea[tiab] OR gonococcus[tiab] OR gonorrhoeae[tiab] OR "Chlamydia"[Mesh] OR chlamydia[tiab] OR "Urinary Tract Infections"[Mesh] OR "urinary tract infection*"[tiab] OR "urogenital infection*"[tiab] OR bacteriuria*[tiab] OR pyuria[tiab] OR "Vaginosis, Bacterial"[MeSH] OR "bacterial vaginosis"[tiab] OR "BV"[tiab] OR "Human Papillomavirus Viruses"[Mesh] OR papilloma*[tiab] OR HPV[tiab] ) |
