# Supplementary material for: Assessing the relationship between menstrual products and reproductive and urogenital tract infections (RUTIs): A systematic review evaluating the evidence and recommendations for future research
Source: PLoS One. 2025 Dec 22;20(12):e0332006. doi: 10.1371/journal.pone.0332006 (PMC12721514; doi:10.1371/journal.pone.0332006)
Supplement: S4 Table — (DOCX) [file pone.0332006.s004.docx]

Supplementary Table 3. Detailed Appraisal of Quality of Evidence

| **Study (First Author, Year)** | **Outcome** | **Evidence for each criterion** | | | |
| --- | --- | --- | --- | --- | --- |
|  |  | **Criterion 1: Product Definition** | **Criterion 2:**  **Comparator Definition** | **Criterion 3: Outcome Definition** | **Criterion 4: Confounder Consideration** |
| Abulizi, 2017 | HPV | ----- | -* | ++ | ++*+*+- |
| AlKarmi, 2024 | UTI; RTI unspecified | --+^§^-- | - | +ª- | -+*-+*+* |
| Baisley, 2009 | BV | +---- | +* | ++ | -++*+- |
| Balamurugan, 2012 | RTI  unspecified | ----- | -* | -*- | -+*-+- |
| Chakrabarty, 2023 | RTI unspecified | +---- | -* | -- | ++-+- |
| Das, 2015 | BV & UTI | +-+++ | +* | ++* | -++++ |
| Das, 2021 | BV & VVC | +++++ | + | ++ | -+++- |
| Demba, 2005 | BV | +---- | +* | ++ | +*-+++ |
| Foxman, 1995 | UTI (first time) | ----- | -* | ++ | +--+- |
| Geiger, 1996 | Candidiasis | ----- | +* | +^§^+* | +*-+++ |
| Hansen, 1985 | GV | ----- | - | ++ | +*---- |
| Howard, 2011 | UTI, Vaginitis | ++--- | -* | -- | +*--++ |
| Janoowalla, 2020 | UTI & VV symptoms | ++*++- | +* | ++* | +*+++- |
| Klatt, 2010 | BV (recurrent) | ----- | -* | + | +-+-- |
| Klebanoff, 2010 | BV | ----- | -* | ++ | ++*++- |
| Leibovici, 1984 | UTI | ----- | -* | ++ | +--+- |
| Madar, 2024 | Urogenital infection | --+^§^+^§^- | - | +- | +*+*-+- |
| Mehta, 2021 | BV, STI | ----- | -* | ++ | ++*+*++ |
| Mehta, 2023 | BV, Trichomonas, Gonorrhea, Chlamydia | +*+*--- | - | ++ | +++*++ |
| Morison, 2005 | BV | +-+-- | -* | ++ | +*--+- |
| Nabwera, 2021 | UTI, BV & Candida | +*-++- | -* | +^§^+* | -+++- |
| Omar, 1998 | UTI, Gonorrhea, Chlamydia, Syphilis, HIV | ----- | -* | +- | +--+- |
| Philip, 2013 | RTI/STI  unspecified | ----- | -* | -- | -+*-+*- |
| Phillips-Howard, 2015 | HIV | ++*--- | -* | ++ | ++-++ |
| Phillips-Howard, 2016 | BV, Candida Albicans, Trichomonas, Gonorrhea, Chlamydia | +*+*--- | - | ++ | +*+-+- |
| Singh, A 2022 | RTI/STI unspecified | --+-- | - | -- | -++*+- |
| Singh, M 2022 | Gonorrhea, Chlamydia chancroid, & HIV | --+-- | -* | +- | -+-+- |
| Tchoudomirova, 1998 | UTI (recurrent) | ----- | -* | ++ | +-+-- |
| Torondel, 2018 | Candida, BV, Trichomonas | ++++*+ | + | ++ | -++++ |
| Unzeitig, 2007 | BV, UTI | ----- | -* | ++ | +---- |
| Zulaika, 2023 | HIV | +*+*--- | + | ++ | +*+*-+- |
| **Product Definition:**  +: Product factor defined. Five factors considered (L to R): i) product type beyond label of “pad” such as commercially made, reusable, disposable, heavy absorbent, panty liner, brand name; ii) product material (e.g. silicone, cotton, nylon, silk, perfumed/non-perfumed etc.); iii) frequency of product change; iv) washing/drying practices for reusable products; v) storage practices for reusable products.  -: product factor not defined  +*: not fully defined (e.g. if multiple products in a study or for washing but not drying), but some information  +^§^: product factor defined/data captured, but not considered in analysis even if found to be significant.  **Comparator Definition:**  +: defined (according to at least the first 3 product factors)  +*: not fully defined, but more information than just product category  -: not defined (e.g. “usual practice” with no further information)  -*: compared defined by product category (e.g. pad) but no further product information  **Outcome Definition:**  ++: outcome(s) defined for all groups & confirmed with laboratory testing (self-testing or trained health worker)  +-: outcome(s) defined for all groups, but no confirmatory laboratory testing used (i.e. symptom self-report)  -+: outcome(s) not defined, but confirmed with laboratory testing (self-testing or trained health worker)  +^§^: outcome defined for some groups (e.g. clinic cases) or outcomes but not others (e.g. population controls)  +*: outcome confirmed with laboratory testing for some study groups or outcomes but not all (i.e. symptoms)  +ª: some outcomes defined (e.g. if multiple outcomes measured) but not all  --: outcome(s) not defined & no confirmatory laboratory testing used (i.e. symptom self-report)  **Confounder Definition:**  +: confounder included. Five categories considered (L to R): i) sexual behaviour(s), ii) socioeconomic status, iii) water, sanitation & hygiene including personal hygiene (e.g. douching, etc.), iv) age, v) recent antibiotic use.  +*: some confounder data captured but limited (e.g. WASH access but not personal hygiene/douching habits) or captured but not considered in analysis even if found to be significant.  -: no confounder data captured | | | | | |
