# Supplementary material for: Assessing the relationship between menstrual products and reproductive and urogenital tract infections (RUTIs): A systematic review evaluating the evidence and recommendations for future research
Source: PLoS One. 2025 Dec 22;20(12):e0332006. doi: 10.1371/journal.pone.0332006 (PMC12721514; doi:10.1371/journal.pone.0332006)
Supplement: Attachment — Submitted filename: Menstrual Health Reviewer Comments to Address.docx [file pone.0332006.s007.docx]

**Menstrual Health Reviewer Comments to Address**

**Deadline:** November 20^th^ 2025

**Changes to our manuscript highlighted within the document by using the track changes mode in MS Word**

| **#** | **Comment** | **Response** |
| --- | --- | --- |
| Editor Comments | | |
| 1 | Please ensure that your manuscript meets PLOS ONE's style requirements, including those for file naming. The PLOS ONE style templates can be found at [main body formatting](https://journals.plos.org/plosone/s/file?id=wjVg/PLOSOne_formatting_sample_main_body.pdf) and [title formatting](https://journals.plos.org/plosone/s/file?id=ba62/PLOSOne_formatting_sample_title_authors_affiliations.pdf) | We confirm that our manuscript meets PLOS ONE’s style requirements including those for file naming. |
| 2 | We note that the grant information you provided in the ‘Funding Information’ and ‘Financial Disclosure’ sections do not match.  When you resubmit, please ensure that you provide the correct grant numbers for the awards you received for your study in the ‘Funding Information’ section.  We note that you received funding from a commercial source: [Hologic Corporation, U.S. National Institutes of Health and the Gates Foundation, U.S. National Institutes of Health] Please provide an amended Competing Interests Statement that explicitly states this commercial funder, along with any other relevant declarations relating to employment, consultancy, patents, products in development, marketed products, etc. Within this Competing Interests Statement, please confirm that this does not alter your adherence to all PLOS ONE policies on sharing data and materials by including the following statement: "This does not alter our adherence to PLOS ONE policies on sharing data and materials.” (as detailed online in our [guide for authors](https://urldefense.com/v3/__http:/journals.plos.org/plosone/s/competing-interests__;!!K-Hz7m0Vt54!k-3J-_x9vLaRaFEvF3_5ZLjDaeiaa4cGHha38jvH3YIY1FxuTeR2CaBO-VoAHYHIHHULR8GXPq9YVBQR$)). If there are restrictions on sharing of data and/or materials, please state these. Please note that we cannot proceed with consideration of your article until this information has been declared. | Thank you for bringing this to our attention. Please find the amendments below.  Financial Disclosure: “*This work was supported by the Gates Foundation as part of the University of Washington START Center grant No. GR018228 (SEH) https://www.gatesfoundation.org/. The funder commissioned the study but did not have control over the design, data collection, analysis, conclusions, or preparation of the manuscript.*”  Competing Interests: “I (RSM) have read the journal's policy and the authors of this manuscript have the following competing interests: *received research support and STI testing supplies, unrelated to the current research, from Hologic Corporation.*  “I (SEH) have read the journal's policy and the authors of this manuscript have the following competing interests: *funded by grants from the U.S. National Institutes of Health and the Gates Foundation.*  I (GKZ) have read the journal's policy and the authors of this manuscript have the following competing interests: have *received a grant from the U.S. National Institutes of Health, unrelated to this work, and have received consulting employment through Boston Scientific Corporation, unrelated to this work.”*  “I (JL) have read the journal's policy and the authors of this manuscript have the following competing interests: I have *received grants from the U.S. National Institutes of Health, unrelated to this work. I am also on the Scientific Advisory Board for and received stock in Prosetta Bioconformatics, Inc.*  Country of Main Funder: United States |
| 3 | Please remove all personal information, ensure that the data shared are in accordance with participant consent, and re-upload a fully anonymized data set. Note: spreadsheet columns with personal information must be removed and not hidden as all hidden columns will appear in the published file. Additional guidance on preparing raw data for publication can be found in our Data Policy (https://journals.plos.org/plosone/s/data-availability#loc-human-research-participant-data-and-other-sensitive-data) and in the following article: http://www.bmj.com/content/340/bmj.c181.long. | Thank you for your careful review and for bringing this concern to our attention. Please note that all names included in both supplemental files correspond solely to the first authors of the studies included in our review, and no other identifying personal information is present. Additional responses below. |
| 4 | We note that there is identifying data in the Supporting Information file <Supplemental_MH_Data_Extraction_.xls, Detailed Quality Assessment.docx>. Due to the inclusion of these potentially identifying data, we have removed this file from your file inventory. Prior to sharing human research participant data, authors should consult with an ethics committee to ensure data are shared in accordance with participant consent and all applicable local laws. Data sharing should never compromise participant privacy. It is therefore not appropriate to publicly share personally identifiable data on human research participants. The following are examples of data that should not be shared: -Name, initials, physical address -Ages more specific than whole numbers -Internet protocol (IP) address -Specific dates (birth dates, death dates, examination dates, etc.) -Contact information such as phone number or email address -Location data -ID numbers that seem specific (long numbers, include initials, titled “Hospital ID”) rather than random (small numbers in numerical order) Data that are not directly identifying may also be inappropriate to share, as in combination they can become identifying. For example, data collected from a small group of participants, vulnerable populations, or private groups should not be shared if they involve indirect identifiers (such as sex, ethnicity, location, etc.) that may risk the identification of study participants. Additional guidance on preparing raw data for publication can be found in our [Data Policy](https://journals.plos.org/plosone/s/data-availability#loc-human-research-participant-data-and-other-sensitive-data) and in the [following article](http://www.bmj.com/content/340/bmj.c181.long). Please remove or anonymize all personal information (<specific identifying information in file to be removed>), ensure that the data shared are in accordance with participant consent, and re-upload a fully anonymized data set. Please note that spreadsheet columns with personal information must be removed and not hidden as all hidden columns will appear in the published file. | Thank you for your careful review and for bringing this concern to our attention. We have thoroughly examined the supplemental files included in our submission and identified the following potential sources of identifying information:   1. In the Supplemental MH Data Extraction spreadsheet, there was a column labeled "Study ID" containing the first author’s last name and publication year. To avoid any potential confusion, we have re-labeled this column as "First Author & Year." Additionally, a column labeled "Reviewer Name," which only contained the term "Consensus" in each row, has been removed. We have also removed the column labeled “Covidence ID” as this information is not necessary. 2. In the Detailed Quality Assessment, two author last names appeared under a table referring to "self-swab." We have removed this note since the information is already adequately captured in the table.   All names included in both supplemental files are those of the first author of the included studies in our review.  If there are other specific instances or concerns regarding identifying information that we may have overlooked, please kindly let us know. We will address them promptly to ensure full compliance with the journal’s policies. |
| 5 | Please include captions for your Supporting Information files at the end of your manuscript, and update any in-text citations to match accordingly. Please see our [Supporting Information guidelines](http://journals.plos.org/plosone/s/supporting-information.) for more information. | We have added the captions for Supplementary Table 3 and confirm that all in-text citations to supplemental files match. |
| 6 | If the reviewer comments include a recommendation to cite specific previously published works, please review and evaluate these publications to determine whether they are relevant and should be cited. There is no requirement to cite these works unless the editor has indicated otherwise. | No reviewer comments received regarding previously published works. |

| **Reviewer #1** | | |
| --- | --- | --- |
| **#** | **Comment** | **Response** |
| 1 | Abstract: The sentence "Results were evaluated and synthesized using tabular methods according to measures of association, and across four criteria categories..." is slightly long and could be broken into two for clarity. Again, please consider stating the number of studies that fully defined products (3.2%) and comparators (9.7%) in the results to mirror the impact of the main text, rather than just the percentages for well-defined (6.5%, 9.7%). | Thank you for this comment we have revised this sentence in the methods and amended the results section of the abstract. |
| 2 | Methods (i) Quality assessment framework: The framework is well-justified and operationalized. However, the "star" scoring system, while intuitive, is described in the footnote of Table 2 rather than in the main methods text. A brief description of the scoring logic (e.g., "Each criterion was scored on a scale of 1-4 stars based on the completeness of definitions...") should be included in the "Quality Assessment and Analysis" section for clarity before the reader reaches the table. (ii) Use of AI: The use of AI (ChatGPT-4) for translation is noted. This is an emerging practice. It would be prudent to add a brief sentence acknowledging this as a potential limitation (e.g., "While AI-assisted translation was used as a pragmatic tool, we acknowledge that it may not capture nuanced language as accurately as a human translator."). This could be placed in the Methods or the Limitations section. (iii) Protocol Registration: The authors state "This review was not registered." It is now considered good practice to register systematic review protocols (e.g., on PROSPERO) to minimize bias. This should be acknowledged as a limitation in the discussion. | i) Brief description added to the methods section: *Each criterion was scored from two to five stars based on the completeness of its definitions across relevant factors*.  ii) Thank you, added to the limitations section: *Fourth, while AI-assisted translation was used as a pragmatic tool, we acknowledge that it may lack the nuanced accuracy of human translation. For the single article where this approach was used the English language abstract to verify key details and carefully reviewed the AI-assisted translation to confirm that the text was coherent and the scientific content was logical; however, we did not independently verify the full translation of the article with a fluent speaker.*  iii) Added to the limitations section*: Fifth, our protocol was not registered with PROSPERO, which would have increased transparency and created an audit trail of our objectives and methodology, including search strategy and inclusion criteria.* |
| 3 | Results (i) Table 2: The table is highly informative but very dense. The footnotes are essential for interpretation but are lengthy. Ensure the journal's formatting allows this critical information to be accessible to the reader. (ii) Synthesis of findings: The decision to narratively synthesize the results from the nine highest-rated studies is appropriate given the heterogeneity. The summary is balanced, highlighting both significant and null findings. The reference to S2 Table for all studies is correct. (iii) Clarity: The results for Criterion 1 (Product Definition) state "only two articles (6.5%) provided partial or full definitions across all four factors." However, in the Discussion, it's stated that "only one article (3.2%) fully defined their menstrual product(s)." This slight discrepancy (full vs. partial/full) should be clarified for consistency. It's a minor point but worth checking. | i) Thank you for raising this. We have confirmed that the journal permits table footnotes.  ii) Thank you.  iii) Thank you for highlighting this. Results revised for clarity to: …*only one article provided partial (3.2%) and one other article (3.2%) provided full definitions across all four factors.* |
| 4 | Discussion: As mentioned above, please add the lack of a pre-registered protocol as a minor limitation. Again, the paragraph on the "hygienic"/"unhygienic" labeling is a very important conceptual point. It could be slightly strengthened by more explicitly stating that such value-laden terms are not only biased but also scientifically uninformative. | i) Thank you we have added a point to the limitations section as noted above.  ii) Thank you we have added an explicit mention of this stating: *Such labels risk reinforcing stigma rather than reflecting empirical findings and are not only biased but also significantly uninformative.* |
| 5 | Limitations: The limitations section is thorough and self-critical, covering the key points: database selection, heterogeneity preventing meta-analysis, and reliance on published data. Additionally, please incorporate the two points mentioned above: 1) the use of AI for translation, and 2) the lack of a pre-registered protocol. | 1) Added to the limitations section as above.  2) Added to the limitations as above. |
| 6 | References: Check reference #8 (OpenAI. ChatGPT-4). While citing AI models is becoming more common, ensure it aligns with the specific citation guidelines of the journal. | No specific formatting guidelines identified but amended to align with [this 2024 PLOS One article](https://journals.plos.org/plosone/article?id=10.1371/journal.pone.0301854) on ChatGPT-4 |
| 7 | Language and clarity: Only minor proofreading is required. For example: Page 11, Line 67: "understandings" could be "understanding". Page 12, Line 92: "assistance of artificial intelligence (AI)" could be "assistance from artificial intelligence (AI)". | Thank you for highlighting these. Corrections made. |

| **Reviewer #2** | | |
| --- | --- | --- |
| **#** | **Comment** | **Response** |
| 1 | The authors state, "This review was not registered" (Page 6, line 134) without any justification. The pre-registration of a systematic review protocol (eg., in PROSPERO) is an important standard of practice. Registration helps prevent reporting bias by pre-specifying the review's objectives, search strategy, inclusion criteria, and analysis plan. The absence of a registered protocol must be explicitly acknowledged and discussed in the limitations section. | Thank you for raising this. We have now acknowledged this in our limitations section: *Fifth, our protocol was not registered with PROSPERO, which would have increased transparency and created an audit trail of our objectives and methodology, including search strategy and inclusion criteria.* |
| 2 | The use of AI for translating articles is an innovative approach. However, this is not yet a standard practice in systematic reviews. The manuscript lacks crucial details on the process used to ensure the accuracy of these translations. The authors should elaborate on their validation process: a) Was the AI-translated text later reviewed by a fluent speaker of the original language? If not, can this be arranged? b) Was a sample of articles professionally translated or reviewed to assess the reliability of the AI tool? Without a clear validation process, the accuracy of the data extracted from these non-English articles remains questionable. | Thank you for raising this important point. We would like to clarify that AI-assisted translation was used for only one article in our review. To help verify the accuracy of key details, this article had an English language abstract which we used to cross-check the translation. However, we did not engage a fluent speaker to independently verify the translation, and we acknowledge this limitation. We appreciate the reviewer’s concern regarding validation and agree that a more formal validation process would strengthen confidence in extracted data from non-English sources. We have updated our methods and limitations accordingly to note:  1. *For the single article with an available English abstract but with our team lacking a member fluent in language of the full text, AI tools (ChatGPT-4) were employed to assist with translation for full-text review*.  *2. For the single article where this approach was used, the English language abstract was used to verify key details and we carefully reviewed the AI-assisted translation to confirm that the text was coherent and the scientific content was logical; however, we did not independently verify the full translation of the article with a fluent speaker.* |
| 3 | The four-criteria quality assessment is a key strength, but its construction requires further justification to enhance its transparency and robustness. a) The authors should provide a rationale for weighting the four criteria (product, comparator, outcome, and confounder definition) equally. It could be argued that flaws in outcome definition (e.g., reliance on self-reported symptoms) pose a more severe threat to a study's validity than a poorly defined comparator product. b) The thresholds for assigning stars within each criterion appear arbitrary. For example, why is the cutoff for the highest score on confounder consideration "≥4 relevant confounders"? (Table 2). A brief explanation for the choice of these cutoffs would strengthen the methodology. | Thank you for highlighting this concern. We have updated our methods as follows:  *We did not include weighting of different criteria in our quality assessment because the purpose was to conduct a descriptive evaluation of study quality rather than to generate a weighted score. This approach aligns with common practices where quality assessments serve to characterize methodological strengths and limitations without imposing arbitrary weights. A full breakdown of the quality assessment results, excluding any cut-offs or summary scores, is available in Supplemental Table 3 for transparency*. |
| 4 | In the "Study Findings" subsection, the authors state they are presenting "a summary of findings from the nine highest quality studies. The manuscript must clearly define how these nine studies were selected. Was a cumulative star score from Table 2 used? If so, what was the threshold for inclusion? This lack of transparency makes it difficult to evaluate the potential for selection bias. | Thank you for this comment. We agree that providing additional clarity on the selection of these nice studies would improve transparency of our reporting. We have updated the first paragraph of the “Study Findings” subsection as follows:  *For simplicity of reporting, the following section summarizes findings from the nine highest-quality studies, selected according to the four criteria in Table 2. Studies meeting an average of three stars per criterion or a total of at least twelve cumulative stars were included. Results from all studies are available in S2 Table.* |
| 5 | The search was restricted to three databases (PubMed, Web of Science, and FDA MAUDE). The authors should provide a rationale for excluding other major health and medical databases as their omission may have resulted in missing relevant studies. | We have addressed this concern more fully in our limitations section.  *First, we only included publications meeting eligibility criteria from three databases (PubMed, Web of Science, and FDA MAUDE). Those indexed only elsewhere would not have been included; however, these databases likely capture most peer-reviewed literature and product safety reports relevant to our search.* |
| 6 | The star rating for confounder consideration provides a good summary but obscures important details. To provide a richer synthesis, the authors should consider a supplemental table that specifies which of the five confounder categories (sexual behavior, SES, WaSH, age, antibiotic use) were addressed in each study, rather than just presenting a summary score. | Thank you for your feedback regarding the limitations of the summarized scores, which is an important point. To address this, we uploaded a supplemental table labeled ‘Detailed Appraisal Quality of Evidence’ with our original submission. This table provides the full breakdown and assessment for each study to ensure readers have access to additional detail beyond the summary table. We have moved this table to the file containing the S1 and S2 tables to make this easier to locate and apologize for any confusion. Please let us know if further action is needed on our end to ensure the full table is available for review. |
| 7 | The authors list the inability to conduct a meta-analysis as a limitation of their review. This is more accurately a key finding of the review that powerfully demonstrates the extreme heterogeneity and poor standardization in the primary literature. It is a failure of the field, not a limitation of this review. This point should be reframed to reflect that. | Thank you for your feedback regarding the reporting of our inability to conduct a meta-analysis. While we had reported this as a finding in the first paragraph of our results section: “*A meta-analysis of outcomes was not possible due to data heterogeneity across the product, comparator, and outcome definitions as shown in Table 2.*” As well as in the abstract and recommendations for future research.  We have now added more explicit mention of this finding to the first paragraph of the discussion section: *The lack of uniform product definitions and comparators does not allow for an aggregation of findings across studies which would have allowed for the conduct of a meta-analysis.* |
| 8 | The authors rightly criticize a study where 82-93% of participants in the "cup group" also used other products. This is an excellent point about exposure misclassification. They should consider elevating this issue in the Discussion as a critical challenge for intervention studies in this field, as concurrent product use is common and complicates the assessment of a single product's effect. | Thank you for your valuable suggestion to elevate the point on challenges related to assessing single-product effects. In our initial submission, we addressed this issue in the first paragraph of the Discussion and further highlighted the importance of precise study group labeling in the second paragraph. To enhance clarity and emphasis based on your feedback, we have now introduced a more explicit statement on these challenges in the first paragraph. Additionally, we have strengthened this point in our recommendations for future research, where we emphasize complexities arising from the use of one or more products. We believe these revisions more clearly foreground the issue throughout the Discussion while maintaining logical flow. We appreciate your guidance in helping us emphasize this important consideration. |
