# Supplementary material for: Assessing the relationship between menstrual products and reproductive and urogenital tract infections (RUTIs): A systematic review evaluating the evidence and recommendations for future research
Source: PLoS One. 2025 Dec 22;20(12):e0332006. doi: 10.1371/journal.pone.0332006 (PMC12721514; doi:10.1371/journal.pone.0332006)
Supplement: Attachment — Submitted filename: Menstrual Health Reviewer Comments to Address_2.docx [file pone.0332006.s008.docx]

**Menstrual Health Reviewer Comments to Address**

**Deadline:** January 1^st^ 2025

**Changes to our manuscript highlighted within the document by using the track changes mode in MS Word**

Reviewer #1 – No comments to address

| **Reviewer #2** | | |
| --- | --- | --- |
| **#** | **Comment** | **Response** |
| 1 | Abstract: The sentence in the Methods, "Results were evaluated and synthesized using tabular methods according to measures of association. This was done across four criteria categories..." is slightly awkward. Consider combining for flow: "Results were evaluated and synthesized using tabular methods according to measures of association, assessed across four criteria categories:..." | Thank you for this comment we have revised this sentence in the methods section of the abstract. |
| 2 | 2. Language and Grammar: The manuscript is well-written and clear. The language is formal and appropriate for a scientific journal. Only very minor proofreading issues were noted, which are likely to be caught in the final copyediting process. (i) Page 11, Line 67: "rigorous understandings" could be changed to "a rigorous understanding" for better flow. (ii) Page 42, Line 94: "assistance fromof artificial intelligence" – the tracked change has left an artifact. This should be "assistance from artificial intelligence". | Thank you for your careful review of our manuscript.   i) This issue was addressed with the first revision. We could not locate this specific problem persisting in either the clean or track changes version of the revised manuscript. However, upon review, we updated line 188 (pg. 10 of the track changes version) from “nuanced understandings” to “nuanced understanding” ii) We could not locate the “fromof” issue in either version and have verified that the updated clean version does not contain this error. |
